# Characteristics of suicide attempts seen in emergency service

**DOI:** 10.1192/j.eurpsy.2023.1832

**Published:** 2023-07-19

**Authors:** R. Ouali, R. Masmoudi, F. Guermazi, A. Mellouli, O. Chakroun, R. Sellami, E. Derbel, I. Feki, J. Masmoudi, N. Rekik

**Affiliations:** 1 Psychiatry A, Hedi Chaker University Hospital; 2 Emergency department, Habib Bourguiba University Hospital, Sfax, Tunisia

## Abstract

**Introduction:**

The suicidal phenomenon constitutes a real public health problem not only by the human losses it causes, but also by the psychological and social problems to which it testifies.

**Objectives:**

The objective was to describe suicide attempts in patients hospitalized in the emergency room.

**Methods:**

A descriptive cross-sectional study was carried out with patients admitted to vital emergencies for attempted suicide over a period of 6 months.

We excluded Patients with major cognitive impairment, which prevents understanding of the questionnaire

A data collection sheet was used for the evaluation of the suicide attempts.

**Results:**

Our sample consisted of 101 suicide attempts. Using non-physical methods (drugs, caustics, pesticides, gases) was reported in 91.9% of cases and while physical methods (hanging, phlebotomy, drowning) in 8.9% of cases. Self-poisoning by medications was the most frequent (51%) method used in suicide attempt. The majority of suicide attempts were reactive (77.2%). Family or marital conflicts were the precipitating factor most mentioned (74%). The suicidal act was unpremeditated in 66% of cases. Communication of suicidal intent either verbally or in writing was reported in 34.7% of cases. The passage to the suicidal act was preceded by taking alcohol in 7% of cases and cannabis in 3% of cases. In 44.6% of cases, regret was the attitude most adopted by suicide attempters towards the act. The somatic state on admission was unstable on the cardiac level in 23% of the cases, on the respiratory level in 27% of the cases and on the neurological level in 38% of the cases

**Conclusions:**

The data from our study suggest that suicidal attempts were mostly unpremeditated. Clinicians should not minimize the significance of impulsive attempts, as they are associated with a similar level of lethality as premeditated attempts

**Disclosure of Interest:**

None Declared

